# Strain-Tuneable Magnetism and Spintronics of Distorted Monovacancies in Graphene

**DOI:** 10.1021/acs.jpcc.2c05494

**Published:** 2022-11-09

**Authors:** Huanyu Zhou, Giuseppe Mallia, Nicholas M. Harrison

**Affiliations:** Department of Chemistry and Institute for Molecular Science and Engineering, Imperial College London, White City Campus, 80 Wood Lane, LondonW12 0BZ, U.K.

## Abstract

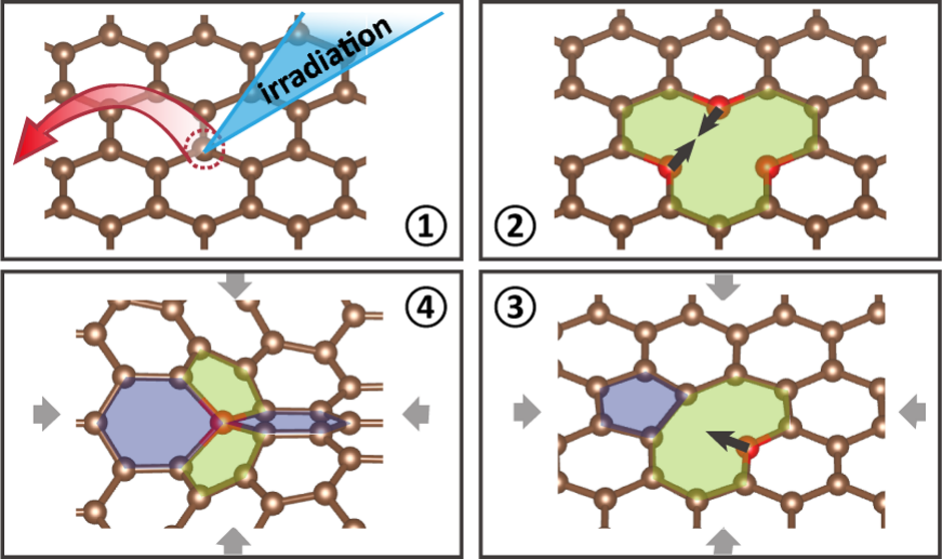

The electronic and spintronic properties of the monovacancies in freestanding and isotopically compressed graphene are investigated using hybrid exchange density functional perturbation theory. When the effects of electronic self-interaction are taken into account, an integer magnetic moment of 2 μ_B_ is identified for a Jahn–Teller reconstructed V_1_(5–9) monovacancy in freestanding graphene. For graphene with stable ripples induced by a compressive strain of 5%, a bond reconstruction produces a V_1_(55–66) structure for the monovacancy, which is localized at the saddle points of the ripple. The sizeable local distortion induced by reconstruction modifies both the geometric and electronic properties of rippled graphene and quenches the magnetic moment of the vacancy due to the sp^3^ hybridization of the central atom. The nonmagnetic V_1_(55–66) structure is found to be stable on rippled structures, with the formation energy ∼2.3 eV lower than that of the metastable distorted V_1_(5–9) structures localized at sites other than the saddle points. The electronic ground state of distorted V_1_(5–9) corresponds to a wide range of fractional magnetic moments (0.50–1.25 μ_B_). The computed relative stabilities and the electronic and magnetic properties of the V_1_(5–9) structures are found to be closely related to their local distortions. This analysis of the fundamental properties of defective graphene under compression suggests a number of strategies for generating regular defect patterns with tuneable magnetic and electronic properties and may, therefore, be used as a novel technique to achieve more precise control of graphene electronic structure for various application scenarios such as transistors, strain sensors, and directed chemisorption.

## 1 Introduction

Graphene is well known for its high carrier mobility,^1,2^ which allows for the fabrication of transistors with tunneling lengths beyond the ultimate limit of silicon transistors (∼10 nm).^3^ In previous research, graphene transistors have shown many characteristics superior to conventional transistors, such as ultrafast room-temperature switching ratio for high-speed electronics^4^ and high sensitivity for selective ion sensing.^5^ The unique properties of graphene transistors are due to the semi-metallic nature, where the half-filled delocalized π band generates relativistic massless electrons at the Dirac point.^2^ However, its gapless nature also limits the on/off ratio of graphene transistors^6−8^ and constrains their applications in devices. By locally breaking the bipartite symmetry of the graphene lattice, defects may facilitate a precise tuning of the electronic and spintronic functionalities of graphene.^9−12^

As the simplest vacancy defect, monovacancy has been widely studied both experimentally^13−16^ and theoretically.^17−20^ The monovacancy is generated by removing a carbon atom from the pristine graphene lattice via irradiation of focused electron or proton beams.^14−16^ Following the generation of the monovacancy with three dangling bonds and *C*_3_ symmetry, a symmetry breaking Jahn–Teller reconstruction (JTR) is observed,^15^ leading to a pentagon-distorted nonagon V_1_(5–9) structure with *C*_2_ symmetry. The monovacancy disrupts the delocalized π band and may lead to unpaired electron density that potentially results in a net magnetization of graphene,^18,19^ making it a potential candidate for use in spintronic devices. However, there are debates over the exact magnetic moment of monovacancy,^18,19,21−25^ and Rodrigo et al.^25^ argued that it is an artifact that originates from the finite-size effects in periodic calculations. Although the overall magnetization is usually regarded as zero when multiple monovacancies exist in graphene,^18,23^ the ferromagnetic hysteresis of proton irradiated graphite is observed at room temperature when an external field is applied.^26^

Considering its two-dimensional (2D) nature, the lattice mismatch between graphene and its substrates, on which it is grown or supported, results in non-negligible strains in practice.^27−29^ Strain effectively tunes the electronic properties of a graphene sheet by changing both its overall and local geometries. Particularly, large in-plane compressive strain, which is beyond the Euler-type buckling limit (−0.7 to −0.8%),^30^ can open an electronic band gap by inducing periodic out-of-plane bending, or ripples.^29^ Strains beyond this limit are experimentally achieved by confining graphene monolayers in a polymer matrix or a diamond anvil cell.^30,31^ However, previous theoretical studies have found difficulties that the prediction of ripple wavelengths is predetermined by the choice of periodic simulation cell.^21,32−37^ The nature of the interplay between monovacancies and strain-induced ripples remains an open question. Some preliminary results have been obtained by either applying tensile strains^22,25^ or under the assumption that the strained graphene sheet is ideally flat.^21^ The most noticeable finding is the strain-induced bond reconstruction of monovacancies, indicating the probable existence of novel strain-tuneable properties. The restoration of the pre-JTR structure with three dangling bonds^21,25^ and a second-order JTR^22^ have both been predicted to be stable under tensile strains. For compressive strains, Santos et al.^36^ used density functional theory in the generalized gradient approximation [GGA–density functional theory (DFT)] calculations to discover a new bond reconstruction of monovacancies in rippled graphene, which quenches the spin polarization and suggests that it is possible to switch on/off the magnetization of graphene by bond reconstruction.

In the current work, the tuneable magnetization of monovacancies in compressed graphene is investigated. Properties of the monovacancy in freestanding graphene are first investigated as a reference. Afterward, the ground state periodic rippled structure of a sheet compressed by 5% in-plane isotropic strain is obtained based on the density functional perturbation theory (DFPT).^38−40^ The distribution of monovacancy formation energies across the rippled sheet is then investigated to locate the thermodynamically favorable sites of the monovacancy. The most stable nonmagnetic V_1_(55–66) structure is identified and studied, while the magnetic properties of monovacancies at other positions are discussed. This investigation clarifies the origin of the inconsistent magnetic moments of a freestanding monovacancy reported in the previous literature.^18,19,21−25^ It re-confirms the existence and the essential stabilizing effect of the V_1_(55–66) bond reconstruction, previously reported by Santos et al.,^36^ now using a more rigorous a priori approach that determines the ripple configurations before adding the defects. The relationship between the local geometry of monovacancies and the magnetic moment of the system is also explored in detail. This relationship may offer a practical way to switch on/off the magnetism of defective graphene by strain engineering. It is also found that novel monovacancy patterns can be engineered by applying compressive strains, enabling the more precise tailoring of graphene transistors.

## 2 Computational Details

The calculations reported here are based on DFT and DFPT as implemented in CRYSTAL17.^41−43^ The HSE06 finite range hybrid exchange functional^44^ is used to minimize the significant self-interaction errors inherent in local and gradient corrected functionals in the broken magnetic symmetry of monovacancy graphene.^23,45−49^ A modified Gaussian basis set for condensed matter calculation 6-21G*, with an optimized exponent (0.26 Bohr^–2^) of the outer sp-type shell and a d-type shell,^50^ is adopted. All-electron DFT calculations are performed, so no shape assumption is made to ionic potential or electron charge density. A 2D periodic boundary condition with an open boundary in the third direction (*z*) is used to model the graphene sheet. The 2D first Brillouin zone of the graphene primitive cell is sampled with a 24 × 24 *k*-point mesh generated by the Monkhorst–Pack method.^51^ This mesh explicitly includes the Dirac point. The sizes of the *k*-mesh for supercell calculations are scaled to achieve consistent *k*-point sampling. Mulliken charge analysis is performed to estimate the atomic charge and spin populations. Owing to the semi-metallic nature of graphene, finite temperature Mermin smearing of the Fermi surface is used,^52^ with a width of 10^–4^ Hartree. The convergence threshold of the total energy in electronic minimization is 10^–8^ Hartree per cell. In geometry optimization using the BFGS algorithm, the convergence criteria are 3 × 10^–4^ Hartree Bohr^–1^ for forces and 1.2 × 10^–3^ Bohr for displacements. The optimized lattice constant is 2.45 Å, which agrees well with that deduced from X-ray diffraction (2.46 Å).^53^

The monovacancy defect studied in this paper is assumed to be charge neutral, consistent with the experimental observation.^15^ Therefore, the formation energy *E*_f_ of a monovacancy is defined as follows
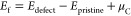
1

*E*_defect_ and *E*_pristine_ are, respectively, the total energies of the defective and pristine graphene; μ_C_ is the chemical potential per carbon atom in the pristine graphene lattice.

To quantify the local curvature of strain-induced ripples, the Laplacian of the atomic *z*-coordinate in a hexagonal lattice, which is summed up to the second nearest neighbors of a given atomic position , can be expressed as^35,54^
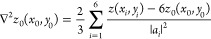
2

*a*_*i*_ is the vector pointing from the central carbon atom at  toward its *i*th second nearest neighbor;  and  , respectively, stand for the *z* coordinates of the central atom and its *i*th second nearest neighbor.

As a result of its nonplanar configuration, the orbital hybridization of carbon atoms in rippled graphene is nonuniformly changed across the sheet and depends on the local geometry. The π orbital axis vector (POAV) analysis is a straightforward parameter to characterize the hybridization of nonplanar conjugated carbon atoms.^55,56^ POAV is defined as the specific vector that makes the three angles, θ_σπ_, between the three *σ* bonds around the central atom and the vector itself equal. The average hybridization *n̅* of σ-orbitals, that is, , may be defined as^56^
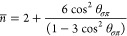
3

## 3 Results and Discussions

### 3.1 Monovacancy

**Figure 1:**
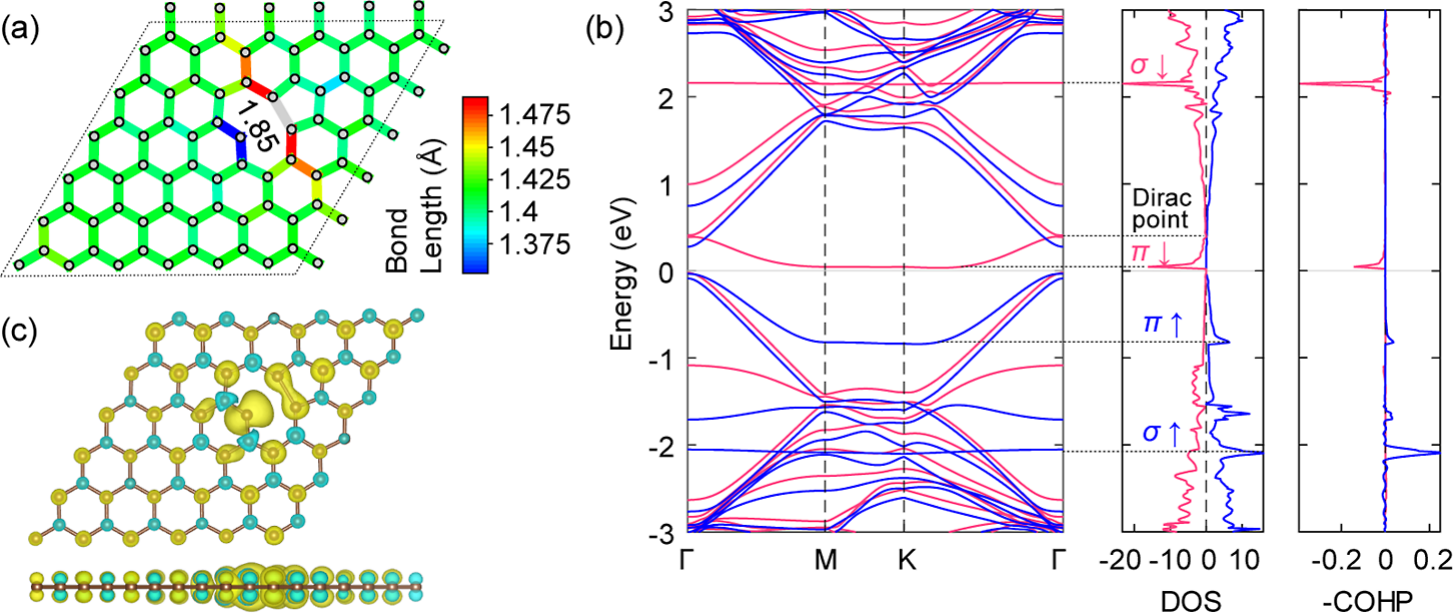
(a) Equilibrium geometry and bond length distribution of a 6 × 6 graphene supercell with a monovacancy. (b) Band structure, the density of states, and reverse crystal orbital Hamiltonian population (−COHP) of defective, freestanding graphene. Blue and magenta lines, respectively, denote spin-up and spin-down states. −COHP curves are projected onto the two atoms connected by the reconstructed bond [plotted in gray in (a)]. The Fermi energy is set to 0. (c) Top and side views of the spin density distribution. Yellow and cyan isosurfaces, respectively, correspond to major and minor spin densities drawn at the value of ±0.02 |*e*| Å^–3^.

The structural, electronic, and magnetic properties of a monovacancy defect in a freestanding 6 × 6 graphene supercell, which corresponds to a defect concentration of 1.4%, are investigated in this section to discuss the origin of its long-debated fractional magnetic moment. The computed formation energy of this monovacancy in freestanding graphene is 8.32 eV, which agrees well with the previous DFT studies on both periodic and isolated systems (7.3–8.58 eV).^24^ In Figure 1a, the relaxed geometry is illustrated and shows that the monovacancy is stabilized by a JTR which leads to a V_1_(5–9) structure with a remaining dangling bond and a stretched C–C bond of 1.85 Å (plotted in gray). The freestanding defective graphene sheet generally maintains its original flat configuration. The elongated reconstructed bond of the pentagon introduces significant local tensile strain that dissipates slowly along the zigzag direction, as indicated by the orange and yellow bonds in Figure 1a, while the two remaining bonds (in blue) of the undercoordinated atom are compressed to increase its effective coordination.

The monovacancy is spin-polarized due to the dangling bond. The calculated magnetic moment of the monovacancy is 2 μ_B_, which comprises one unpaired electron from the dangling sp^2^ bond and 1/3 of an electron from each 2p_*z*_ orbital of the 3 neighbors of the monovacancy.^23^ The computed magnetic moments of a monovacancy in periodic systems have frequently been reported as fractional values between 1 and 2 μ_B_ in previous studies.^18,21,22,57^ This was considered to originate from the finite size effects in periodic calculations and Rodrigo et al.^25^ suggested that the magnetic moment of an isolated monovacancy converges to 2 μ_B_ using large-scale DFT up to 30 × 30 supercell. However, the current study suggests that an alternative explanation for the inconsistency lies in the well-known electron self-interaction error of DFT, which is significant for the previously used (semi-)local functionals based on local spin density approximation (LSDA) and GGA.^45,47,58^ The consequences of this error have been widely documented in strongly correlated systems resulting from the electron confinement that occurs in dangling bonds or n-doped systems.^23,59−62^ Pisani et al.^45^ estimated a significant Hubbard *U* parameter of ∼3.25 eV for graphene ribbons with the GGA–PBE functional, indicating the large self-interaction that exists in the nonbonding localized orbitals when the π band of graphene is disrupted. For the current system, LSDA and GGA functionals may provide a spuriously delocalized charge distribution, leading to incorrect electronic and magnetic ground states. With the range-separated HSE06 functional, the description of the strong on-site Coulomb interactions is improved,^63^ and the magnetic moment of the monovacancy converges to 2 μ_B_ within a relatively small supercell. Therefore, it can be concluded that the self-interaction correction of HSE06 reproduces the same fundamental descriptions, as those provided by global hybrids^47^ and more recently developed hyper-GGA functionals.^58^

The computed band structure and density of states (DOS) of the monovacancy are plotted in Figure 1b. The flat localized defect states, σ and π band, are observed. The majority spin (up) defect state lies on the valance band maximum (VBM), ranging from −0.84 eV to the Fermi level and corresponding to the extended occupied states below the Fermi level. The minority spin (down) π band at conduction band minimum does not cross the Fermi level, and the DOS is 0 at Fermi energy. This differs from the electronic structure reported in some previous studies,^18,21,25^ with the discrepancy due to the correction for electronic self-interaction included here. The monovacancy and resultant magnetic ordering break the symmetry of graphene, opening a band gap of 0.31 eV for majority spin states (spin-up) and 0.06 eV for minority spin states (spin-down). From this, it is clear that if the spins of the monovacancies are aligned, the sheet will be a narrow band gap semiconductor with strongly spin-dependent transport. In the presence of magnetic defect states, the self-doping of the filled valance band upshifts the Dirac point in the spin-down band structure by 0.41 eV. Electrons move from the initially occupied spin-down states near the Fermi surface to the spin-up defect π states.

To characterize the interaction strength of the JTR bond, the spin-polarized COHP curves projected on the two atoms connected by it are plotted in Figure 1b, in a routinely adopted reverse manner (−COHP).^64^ Results suggest that all the spin-up electrons contribute to the bonding states and are beneath the Fermi level, while all the spin-down electrons contribute to the antibonding states and are above the Fermi level. Spin-up states, therefore, stabilize the reconstructed bond. It further supports the conclusion that spin-down π states do not cross the Fermi level. Otherwise, electrons will occupy the antibonding states near the Fermi level, leading to a fractional magnetic moment and reducing the stability of the reconstructed system.

In Figure 1c, the local spin density around the monovacancy oscillates between the graphene sublattices due to the exchange spin-polarization effect. Previous theoretical studies^18,23^ have revealed that the ferromagnetic coupling between the spin localized at the vacancies originates from the regular arrangement of monovacancies in the same sublattice. An antiferromagnetic coupling is generated if monovacancies are accommodated in the opposite sublattices.

Typically, monovacancies in graphene will distribute randomly between the two sublattices and therefore the net macroscopic magnetization will be zero.^23^ Experimentally, monovacancies have been generated in graphite using proton irradiation and aligned in an external field with some ferromagnetic hysteresis observed at room temperature.^26^ This is consistent with the high Curie temperatures predicted for idealized theoretical models of the ferromagnetic phases.^18,23^

### 3.2 Strain-Induced Ripples and V_1_(55–66) Reconstruction

In order to understand the interplay between ripple formation and monovacancies, we first consider the structure of the low energy ripples. DFPT^38−40^ is used to compute the harmonic vibrations of the sheet and, thus, to predict the wavelengths of the low energy strain-induced ripples at 5% isotropic compressive strain. In what follows, positive values denote compressive strains, unless stated otherwise. The wavelength of strain-induced ripples λ can be predicted by the wavevector ***k*** corresponding to the minimum frequency of the soft phonon modes following the relationship of λ = 2π/|***k***|.^40^ The wavevector ***k*** = (1/3π, 1/3π) is found to be near the minimum frequency (Supporting Information) computed and thus the predicted stable ripples are commensurate with a 6 × 6 supercell of the primitive graphene cell. Within this cell, the harmonic ripple can be induced and then the internal coordinates optimized to find representations of the stable ripples. Larger ripple wavelengths are predicted under smaller strains (Figure S1). To ensure the overall reliability of the current study while keeping the simulation cost feasible, the case of the 6 × 6 supercell compressed by 5% strain is investigated in detail, since similar geometries and behaviors are predicted under lower strains with longer wavelengths.^39,40^

**Figure 2:**
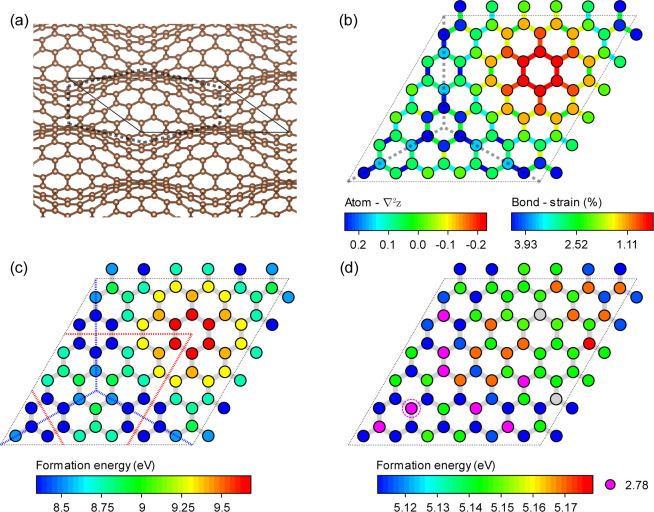
(a,b) Configuration of “egg-box” ripples compressed by 5% isotropic strain. The dashed lines denote the boundary and periodicity of ripple patterns. The solid lines denote the periodic boundary for simulation. In (b), distributions of local curvature (the color gradient on atoms) and local strains (the color gradient on bonds) are given. Strains are calculated based on the equilibrium bond length (1.42 Å). (c,d) Monovacancy formation energy distribution across the 5% compressive strain tuned 6 × 6 supercell before (c) and after relaxation (d). Formation energies are calculated with the rippled pristine cell as the reference. Red and blue dotted lines in (c), respectively, mark the periodic directions of major and minor ripples. Gray atoms indicate that no stable electronic structure was found for that atomic configuration.

The optimized geometry is illustrated in Figure 2a,b. Its stability is confirmed by the absence of imaginary frequency at Γ point. Under isotropic strain, the minimum energy geometry is found to be sensitive to the wavelength of the ripple, but insensitive to its direction in the sheet. A number of low energy ripple patterns are therefore possible and have the general form of a so-called “eggbox” structure with the nonuniformly distributed distortions perpendicular to the 2D lattice plane.^39,40^ Strain-generated ripple patterns show a sixfold symmetry, consistent with the graphene lattice; see Figure 2a. The pattern, as indicated by the gray dashed lines in Figure 2b, consists of an isolated more prominent convex region (major ripple) surrounded by six interconnected flatter concave regions (minor ripple). The major and minor ripples are separated by inflexion points with zero local curvature. The bond strains in this structure are generally smaller than 5% and suggest a significant strain relief through the formation of the ripples. The distribution of the bond strain is inhomogeneous, concentrating on minor ripples and relieving at major ripples. The minimum bond strain (∼0.3%) is observed at the top of the major ripple (see red bonds and red atoms, respectively, in Figure 2b). Around the minor ripples, there are three highly strained bonds (∼4.2%) alternating with the three bonds which are less strained (∼1.5%). This suggests that the interconnected pattern of minor ripples may disperse the compressive strain along the major ripple–minor ripple direction while maintaining the strain concentration along the minor–minor direction.

To document the intricate interplay between monovacancy formation and strain-induced ripples of graphene, the monovacancy formation energy is computed independently at each atomic position in the rippled sheet. For each, considering the experimental procedures to generate monovacancies,^14−16^ the formation energies before and after bond reconstruction are computed. In this way, the energy surfaces, respectively, characterizing the kinetics (i.e., before reconstruction, Figure 2c) and thermodynamics (i.e., after reconstruction, Figure 2d) during monovacancy formation are sampled across the 6 × 6 supercell. In Figure 2c, the energy of removing an atom from the rippled pristine lattice is color-coded, ranging from 8.3 to 9.7 eV. The formation energies of monovacancies in high-curvature regions are larger than that in the low-curvature regions. Comparatively, monovacancies around saddle points (i.e., the intersection points of blue and red dotted lines in Figure 2c) have lower formation energies. The relatively large formation energies in high-curvature regions can be explained by the enhanced coupling of π-orbitals.^33^ This coupling is more prominent at the major ripple where the largest local curvature exists, leading to the highest formation energy.

In Figure 2d, when the geometry optimization of the atomic coordinates is performed, the formation energies (<5.2 eV) at all atomic sites decrease significantly, evidencing the essential stabilization of bond reconstruction. It has to be noted that the monovacancy formation energy in the unstrained flat graphene is 8.32 eV, indicating that the monovacancies are formed more easily in rippled graphene (upon bond reconstruction) and that they provide a strain relief mechanism. This also originates as the compressive strain weakens the sp^2^ σ bonds and localizes the π band (Figure 1b) by deforming the geometry. In Figure 2d, the monovacancy formation energy at the atomic sites shown in magenta is ∼2.78 eV, which is ∼2.3 eV lower than that of the monovacancies at the other sites. Generally speaking, as with the formation energies computed at unrelaxed geometries, the energetically favorable sites locate at the saddle points. However, only the atomic sites simultaneously at the boundaries of the ripple pattern (gray dashed lines in Figure 2a,b) and at the saddle points are characterized by the strong stabilizing effect for the monovacancy formation, with two asymmetric exceptions that are discussed in the next section. For other sites, the formation energies vary with a negligible difference of ∼0.07 eV and are significantly larger than the formation energies at the lowest energy sites. Interestingly, the stabilizing effect of saddle points reported here is consistent with the GGA-based, a posteriori study by Santos et al.*,*^36^ where the energy favorable sites of monovacancies were obtained by first straining the defective graphene sheet and then adding back the missing atom and re-optimizing the geometry.

**Figure 3:**
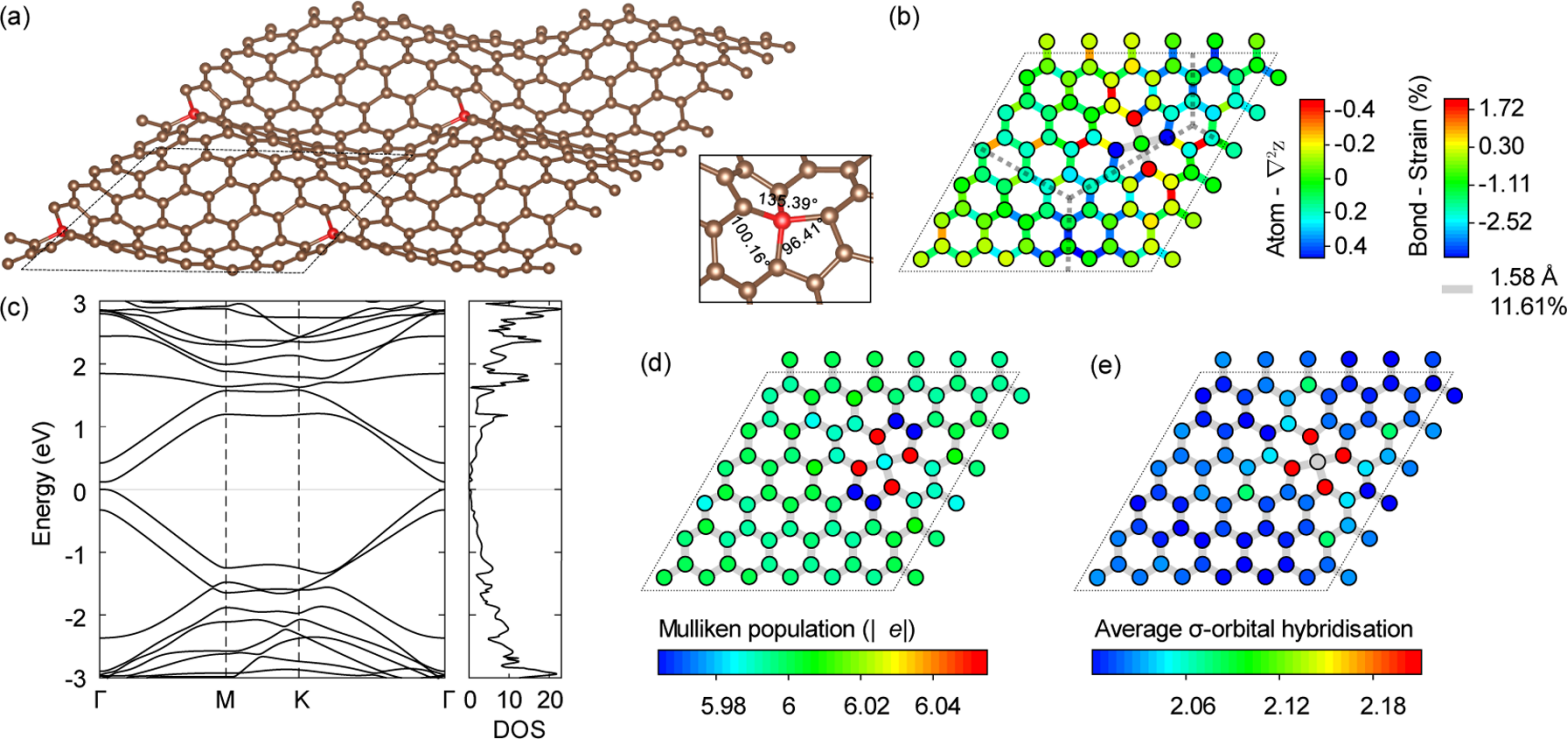
(a,b) Geometry of rippled the 6 × 6 graphene supercell with reconstructed V_1_(55–66) at the saddle point (marked by the dotted magenta circle in Figure 2d). The sp^3^ hybridized atom is marked in red in (a). The inset shows the local geometry around the defect. In (b), positive values denote tensile strains, while negative values denote compressive strains. Gray dashed lines indicate the boundaries of the previous isotropic ripples. (c) Band structure and DOS of the defect system. Fermi energy denoted by solid horizontal gray lines is set to 0. (d) Atomic charge distribution obtained by Mulliken analysis. (e) Distribution of average σ-orbital hybridization. POAV analysis does not apply to the sp^3^ hybridized atom of V_1_(55–66).

A detailed analysis of the reconstructed monovacancy at the saddle point marked by the dotted magenta circle in Figure 2d has been performed to explore its local configuration and properties, since the magnetism at this site is found to be quenched with a magnetic moment equalling 0 μ_B_, indicating the absence of the unpaired electron and the probable existence of a new bond reconstruction. Monovacancies at other energy-favorable sites have similar structures and may be expected to have similar properties. In Figure 3b,d,e, the origin of coordinates has been modified to place the defect closer to the center of the supercell. The supercell boundary used for the calculation is marked in Figure 3a. Figure 3a,b illustrates that, when compressed by 5% strain, the unsaturated carbon atom moves toward the reconstructed bond, which is the transitional configuration in monovacancy migration.^20^ However, in this case, the unsaturated atom is stabilized by an sp^3^ reconstruction and bonded with its four neighbors. Consequently, a reconstructed structure involving two pentagons and two stretched hexagons, V_1_(55–66), is shown in the inset. The central atom in red is sp^3^ hybridized, leading to a slightly flattened tetrahedron and the long sp^3^ bonds with four neighboring atoms. V_1_(55–66) is significantly distorted due to the compressive strain, as characterized by its local curvature. The twisting angle between the opposite pentagons is 52.23°, while the angle between opposite hexagons is 54.40°. The dihedral angles of the neighboring pentagons and hexagons are around 139.66∼139.95°.

Before the V_1_(55–66) monovacancy is generated, hexagons at saddle points bear the largest local strain (Figure 2). By comparing Figures 3b to 2b, the asymmetric nature of the local reconstruction breaks the isotropic symmetry of the strain and ripple distribution across the sheet. Local strains along the hexagon–hexagon direction are largely relieved by the displacement of the central atom of V_1_(55–66), which results in the elongation of some bonds along this direction. Similar strain relief is not observed along the pentagon–pentagon direction, which is vertical to the displacement. Therefore, among the original saddle points, only those along the pentagon–pentagon direction continue to be the regions of strain concentration. The connection between strain distribution and the formation energy of a site indicates the possibility of mediating the monovacancy patterns in the sheet via strains.

The change in local geometry is accompanied by a change in the electronic structure. The band structure and DOS of the ground state configuration shown in Figure 3c reveal an electronic structure that is intermediate between pristine graphene^1^ and an unstrained defective graphene sheet (Figure 1b). The system is found to be nonmagnetic, which is reasonable given that no dangling bond remains in the reconstructed structure. The quench of magnetism is also reported in the GGA–DFT study on strained monovacancy^36^ and the experimental observation of Fe single atom doped divacancy (Fe@DV).^16^ Meanwhile, influences of the defect are observed. Partially localized defect states are observed, which is not as evident as in the case of V_1_(5–9). In contrast to Figure 1b, a spin symmetric band gap is opened in the presence of V_1_(55–66), owing to the bond reconstruction.

The atomic charge population distribution is illustrated in Figure 3d. A charge accumulation region is observed around the central atom of V_1_(55–66). Its second nearest atoms from pentagons transfer the most charge to its nearest neighbors, where the largest distortion is observed. This phenomenon might be caused by the strong σ-orbital coupling originating from the short bonds of pentagons. The charge depletion region is not as obvious, but more extended, along the hexagon–hexagon direction.

In Figure 3e, the average σ-orbital hybridizations () of carbon atoms in the supercell, except the central atom of V_1_(55–66), are plotted. The largest values are reported at the nearest neighbors of the central atom due to local distortions, indicating a greater contribution of sp^3^ hybridization. *n̅* of other atoms surrounding V_1_(55–66) also exhibit a moderate increase. Atoms in rippled regions have much smaller values when compared with those around V_1_(55–66), while atoms at inflexion points maintain the pure sp^2^ hybridization.

**Figure 4:**
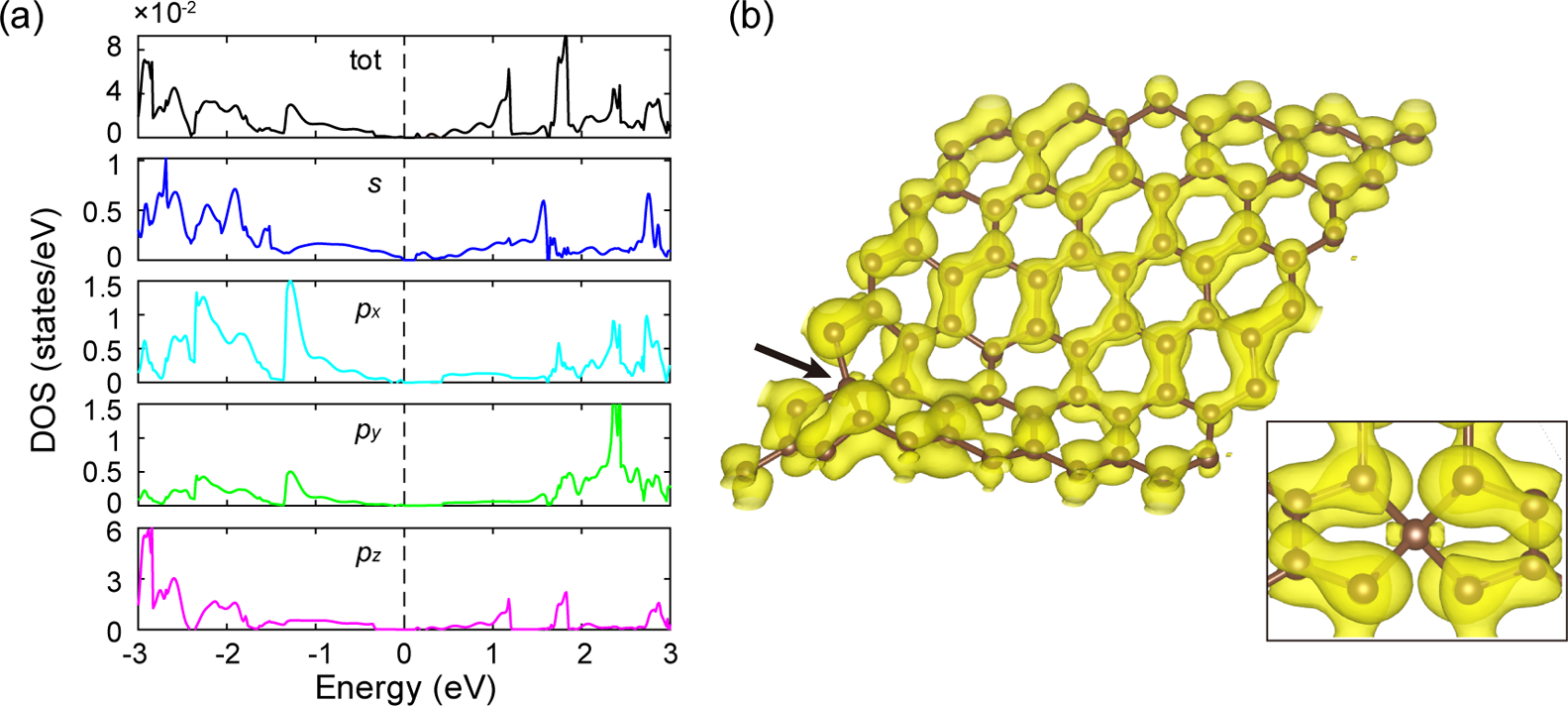
(a) PDOS curves projected on the central atom of V_1_(55–66). Fermi energy denoted by black dashed lines is set to 0. (b) Partial charge density projected on the VBM of the system. The value of isosurfaces is 0.006 |*e*|Å^–3^. The inset shows partial charge density around the central atom of V_1_(55–66). The atom pointed at by the black arrow is the central atom of V_1_(55–66).

The central atom of V_1_(55–66) is bonded with four neighbors; in this case, a pure sp^3^ hybridization should be expected; however, there is a degree of sp^2^ hybridization, considering the asymmetry of the tetrahedron, as shown in Figure 3a. This mixing is evident in the projected DOS (PDOS, Figure 4a) of *p*_*z*_ orbital, which differs from the curves of the other orbitals, especially in the ±1.5 eV region around the Fermi level. Interestingly, compared with those of *p*_*x*_ and *p*_*y*_ orbitals, the PDOS curve of s orbital shows more patterns similar to that of p_*z*_ orbital, including the absence of peaks in energy ranges of −1.5 to −1 eV and 2 to 2.5 eV, and the peaks at ∼1.2 eV, which indicates the more significant mixing between *s* and *p*_*z*_ orbitals.

In the flat graphene, the states at VBM are occupied by electrons from the delocalized π-bonded *p*_*z*_ orbitals.^33^ The partial charge density projected on the VBM of graphene is shown in Figure 4b. The charge depletion region is observed around the central atom of V_1_(55–66) due to its σ-bonded *p*_*z*_ orbital. However, the inset shows an almost negligible charge distribution around it, verifying the conclusion that sp^2^ hybridization also has a slight contribution. Meanwhile, an enhanced π-orbital coupling region is observed around the central atom, corresponding to the charge accumulation region in Figure 3d.

### 3.3 V_1_(5–9) on Rippled Graphene

**Figure 5:**
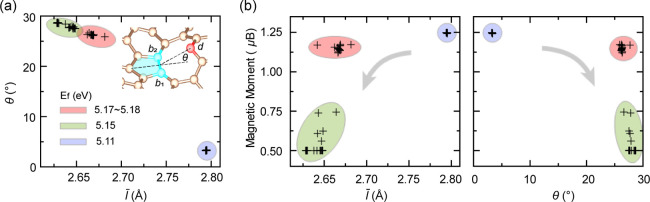
(a) Correlation between the average length *l̅* and the dihedral angle θ. The inset shows the definition of the parameters. (b) Correlations of *l̅* and θ with the magnetic moment. The color-shaded regions indicate the monovacancy formation energy *E*_f_. Local distortion of V_1_(5–9) increases along the gray arrows.

The JTR leads to a pentagon-distorted nonagon V_1_(5–9) structure, which is metastable when the removed atom is not at a saddle point, except for 2 asymmetric V_1_(55–66) cases (Figure 2d). Since V_1_(5–9) structures are found to be metastable with various distortions, they provide ideal cases for discussing the properties of compressive strain-mediated V_1_(5–9) defect. Magnetic moments vary between 0.50 μ_B_ and 1.25 μ_B_ for distorted V_1_(5–9) sites across the 6 × 6 supercell. To understand the origin of these fractional magnetic moments and the stability of distorted V_1_(5–9) on rippled graphene, a statistical analysis of their local geometries is conducted. θ is defined as the dihedral angle between the pentagon and the plane of atoms *b*_1_*, b*_2_ and *d*. l*®* is the average length between *d* and *b*_1_*, b*_2_ (Figure 5a inset). Spearman’s rank correlation coefficients (*r*_S_) are calculated between local geometry parameters and magnetic moments of the distorted V_1_(5–9), as shown in Table 1.

**Table 1:** Spearman’s Rank Correlation Coefficients *r*_S_, Respectively, of the Local Curvature of the Removed Atom ∇^2^*z*_rm_, the Local Curvature of the Undercoordinated Atom ∇^2^*z*_d_, the Dihedral Angle θ, and the Average Distance *l̅* with Magnetic Moments

parameter	∇^2^*z*_rm_^a^	∇^2^*z*_d_	θ	*l®*
*r*_S_	0.53	0.89	–0.97	0.91

a Local curvature before the monovacancy is generated. If not indicated, the monovacancy is present.

Table 1 shows the local curvature of the removed atom ∇^2^*z*_rm_ does not strongly correlate to the magnetic moments. Parameters describing the unsaturated atom *d* show stronger correlations, which is reasonable considering that the atom *d* contributes to the unpaired electron. The parameters θ and *l̅* exhibit strong correspondence (). The correlations among θ, *l̅* and magnetic moments are given in Figure 5. As the distance *l̅* between the unsaturated atom *d* and the JTR bond decreases, the diffuse, unbonded electron cloud experiences increasing Coulomb repulsion. Consequently, the out-of-plane displacement of atom *d*, characterized by θ, increases, enhancing the hybridization between its *p*_*z*_ orbital and sp^2^ hybridized orbitals and gradually reducing the magnetic moment of the whole system. The tuning effect of the local curvature (in the form of the dihedral angle between neighboring aromatic rings) on the overall spin distribution is also recently observed in 2D covalent organic frameworks, which is proven to be robust at a finite temperature.^65^ Even though the monovacancy formation energy *E*_f_ is not monotonically correlated to the local geometry parameters or the magnetic moment of the distorted V_1_(5–9), Figure 5 reveals that V_1_(5–9) monovacancies with similar structural parameters tend to possess similar magnetic moments and formation energies.

Considering the similarities mentioned above, V_1_(5–9) in the same color-shaded region are assumed to possess similar electronic and spintronic properties. To better understand the influences of different local geometries on the electronic structures and stabilities of V_1_(5–9), three cases, respectively, from the blue (low *E*_f_, small distortion), green (medium *E*_f_, large distortion), and red (high *E*_f_, medium distortion) regions are selected.

**Figure 6:**
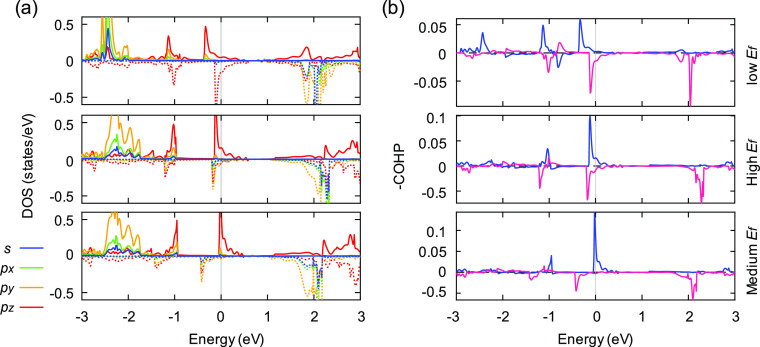
(a) PDOS curves projected on the unsaturated atom. Solid and dotted lines, respectively, denote majority and minority spin states. The DOS range is limited to ±0.6 states/eV. (b) −COHP curves projected onto the two atoms connected by the JTR bond. Blue and magenta lines, respectively, denote majority and minority spin states. Fermi energy is set to 0. Figures are arranged in an ascending order by the local distortion.

Figure 6 illustrates the electronic structures of V_1_(5–9) with various distortions. PDOS curves (Figure 6a) projected on the unsaturated atom, especially in the ±0.1 eV region, reveal an enhanced coupling between the p_*z*_ orbital and the sp^2^ hybridized orbitals as local distortion increases. Consequently, the magnetic moment of the defect system decreases, which explains the statistical trend in Figure 5. Considering the increased bonding states and the decreased antibonding states near the Fermi level, −COHP curves (Figure 6b) suggest that the increased local distortion stabilizes the V_1_(5–9) structure and reduces the probability of reconstruction, which is counterintuitive since V_1_(5–9) with increased distortion has a lower magnetic moment and seems to be closer to the reconstructed V_1_(55–66). However, it is noteworthy that the distortion also disperses the bond strains across the sheet (Figure S2), while the confined geometry and severe strain condition at saddle points probably limit the displacements and keep the strains concentrating at the saddle point, which leads to the V_1_(55–66) reconstruction. Similarly, V_1_(55–66) deviating from saddle points (Figure 2d) are both confined by the ripples nearby, which increases the probability of reconstruction.

**Figure 7:**
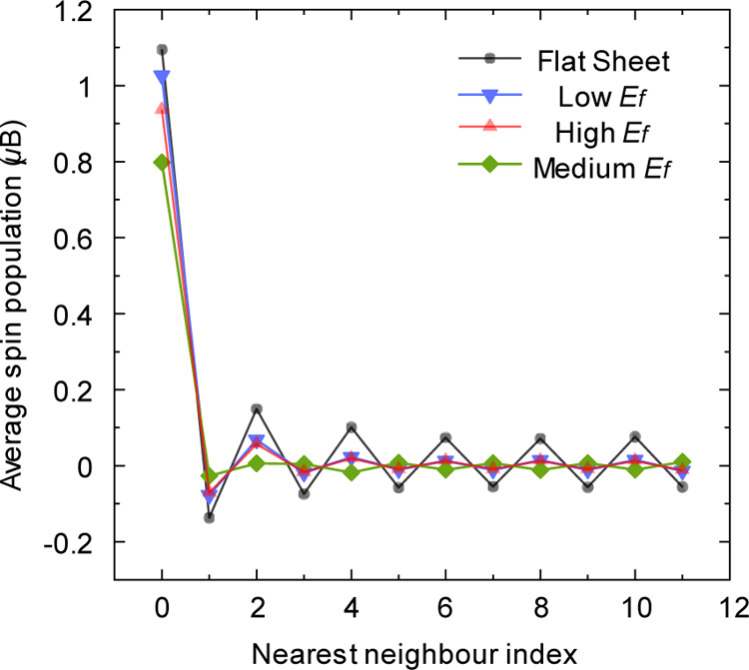
Evolutions of the Mulliken spin population averaged over the *i*th nearest neighbor of the unsaturated atom.

The damped fluctuation patterns illustrated in Figure 7 identify the regular distribution of spin densities across the supercell, which originates from the exchange spin-polarization effect.^18^ This effect is weakened as the distortion of V_1_(55–66) increases due to the stronger sp^3^ hybridization of the unsaturated atom. The curve of the most distorted V_1_(55–66) with medium *E*_f_ shows a reverse spin distribution from the third nearest neighbor, which originates from the reversed spin orientations of the 2 atoms connected by the JTR bond (Figure S3).

## 4 Discussion and Conclusions

The strain-tuneable magnetism of defect graphene is achieved by the crucial interplay between monovacancies and strain-induced ripples. The unstrained V_1_(5–9) monovacancy is predicted to have a spin asymmetric conductivity and localized strong ferromagnetic coupling, with a magnetic moment of 2 μ_B_ when the electron self-interaction error is corrected. A periodic “eggbox” ripple pattern generated by 5% in-plane isotropic compressive strain is predicted to minimize the finite size effects inherent in ad hoc choices of supercells. This study finds that the reconstructed V_1_(55–66) at saddle points of the ripples is the most stable configuration, which forms sp^3^ bonds and quenches the local magnetic moment, with respect to the distorted V_1_(5–9) observed at the other sites across the rippled sheet. Correlations among local distortions, electronic, and spintronic properties, and stabilities of distorted V_1_(5–9) are discussed. It is found that more distorted V_1_(5–9) defects tend to be more stable and have smaller magnetic moments. According to the current study, the switching between spin-polarized and non-spin-polarized states of graphene is achievable by the V_1_(55–66) reconstruction. It can be estimated that this reconstruction does not strongly depend on defect density, since only the local distortions around a monovacancy determine its stability.

In general, by controlling the local distortion of a monovacancy via compressive strains, either a kinetically stable V_1_(5–9) or a thermodynamically stable V_1_(55–66) structure can be obtained. Considering that the substrates are commonly used to generate isotropic strains in graphene, the practical implementation of this mechanism will depend on finding suitable substrates with moderate coupling with graphene, upon which the graphene can be strained while being free from the graphene–substrate interactions that might interfere in the formation of monovacancies or quench magnetic states. As reported by Zhang et al.*,*^66^ structural and spintronic properties of V_1_(5–9) on the rotationally misaligned topmost layer of chemical vapor deposited graphite is well preserved and might be a potential candidate for experimental investigations.

Besides, the stability and diffusion of the generated monovacancy at finite temperatures are still unclear due to the complicated monovacancy migration mechanisms observed in experiment^15^ and the influences of thermal fluctuation on ripple configurations. However, it is still noteworthy that the significant stabilizing effect (∼2.3 eV lower in formation energy) of the V_1_(55–66) reconstruction makes it thermodynamically favorable for a V_1_(5–9) to diffuse toward a saddle point and to get reconstructed at room temperature if the switching mechanism is followed as usually assumed in theoretical studies.^20,57,67^ The hopping barrier decreases as the compressive bond strain increases,^57^ probably making the diffusivity of V_1_(5–9) commensurate with the rippled geometry of compressed graphene. This probably leads to a patterned monovacancy distribution with long-range periodicity tuned by compressive strains, allowing for even further modifications of graphene-based electronics.

Meanwhile, it is also probable for multiple monovacancies to coalesce around the saddle points, where the strain concentrates, leading to the expansion of the vacancy and the failure of the sheet. On the other hand, neighbors around the central atom of V_1_(55–66) exhibit the predominant increase of the frontier (i.e., VBM) electron density (Figure 4b), which, according to the frontier orbital theory, indicates an increased chemical reactivity around V_1_(55–66) defects. This might enable patterned adsorbate self-assembly and functionalization at the molecular level, which is important for the controlled engineering of the configuration and properties of graphene.^40^

In conclusion, via the interplay between ripple and monovacancy, the electronic and spintronic properties of graphene can be effectively tuned to achieve more precise control of graphene, making it a potential option for application in various scenarios.

## Supporting Information Available

A brief explanation of estimating ripple wavelengths based on phonon dispersion curves, and further information about the structural and electronic properties selected cases (PDF)
