# Supplementary material for: Strain-Tuneable Magnetism and Spintronics of Distorted Monovacancies in Graphene
Source: J Phys Chem C Nanomater Interfaces. 2022 Nov 9;126(45):19435–45. doi: 10.1021/acs.jpcc.2c05494 (PMC9677494; doi:10.1021/acs.jpcc.2c05494)
Supplement: Supplementary file 1 — jp2c05494_si_001.pdf [file jp2c05494_si_001.pdf]

Supporting Information for "Strain-tuneable Magnetism and Spintronics of Distorted Monovacancies in Graphene"

Huanyu Zhou*, Giuseppe Mallia, Nicholas M. Harrison

Department of Chemistry and Institute for Molecular Science and Engineering, Imperial College London, White City Campus, 80 Wood Lane, London, W12 0BZ, United Kingdom

* *E-mail address:* huanyu.zhou20@imperial.ac.uk

1. Estimating ripple wavelengths

To eliminate the influences from periodic boundary conditions, the size of the supercell should be commensurate with the ripple wavelengths. The density functional perturbation theory (DFPT) based technique estimates the wavelengths of strain-induced ripples with the phonon dispersion curves of a compressed flat graphene primitive cell. The energetically favourable ripple wavelengths λ correspond to the minimum of soft phonon modes $\boldsymbol{k}$, where the most prominent stabilisation effect can be obtained [1]. When 0% ~ 5% isotropic compressive strains are applied, the phonon dispersions of the graphene primitive cell maintaining the original planar configuration are plotted in **Figure S1**. The planar configuration is stable when no strain is applied. The out-of-plane acoustic (ZA) branch is softened near Γ and the k vector corresponding to the minimal frequency increases as the strain increases, indicating the growing harmonic instability of the planar structure and the reduced ripple wavelengths.

A ripple pattern periodic along the armchair direction is chosen for study in the main text. In practice, due to the isotropic strain, 'eggbox' structures are found to be nearly degenerate in energy with a range of similar rippled structures having the same wavelength and different orientations. In **Figure S1**, the minimum of softened ZA branch corresponds to an optimal supercell size of 5×5. However, considering the flat bottom of the imaginary ZA branch (**Figure S1**) and the band folding in the supercells (to ensure that the Dirac point is accurately sampled), in **Table S1** phonons and ripple formation energies corresponding to 5×5 and 6×6 supercells are compared, showing no significant change in the reported frequencies.

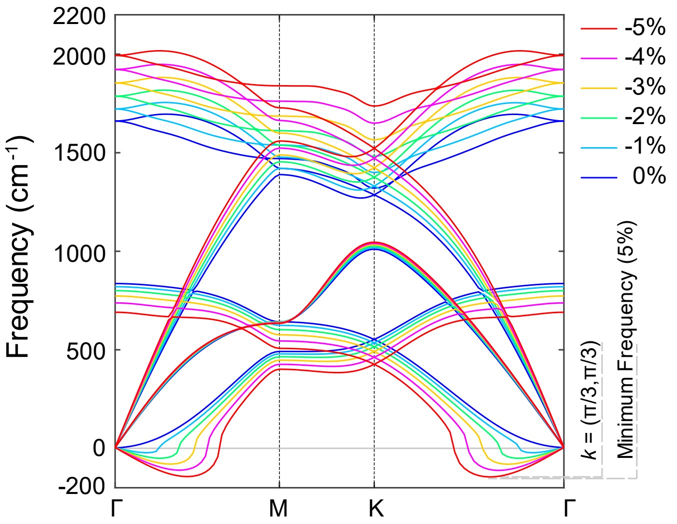

Figure S1 Phonon dispersion of flat graphene primitive cell compressed by 0% ~ 5% strains. Grey dashed lines indicate the wavevectors respectively corresponding to the minimum frequency and $\lambda = 6$ along the armchair direction.

Table S1 Phonons of 5×5 and 6×6 supercells, 5% strain compressed graphene

Supercell size	$\boldsymbol{k}$	λ	Frequencies (cm^{-1})	Ripple formation energy (eV / C atom)
5×5	(9/40, 0)	4.44	-147.38	-0.05
6×6	(7/40, 0)	5.71	-137.83	-0.08

2. Structural and electronic properties of distorted V_1(5-9)

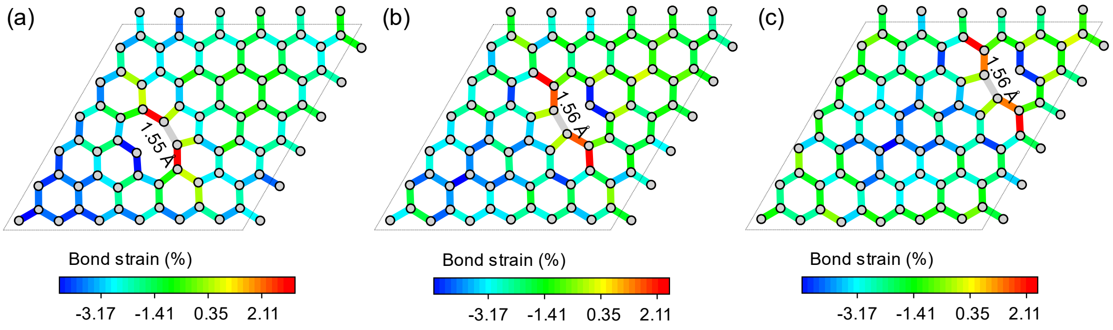

Figure S2 Bond strain distributions of distorted V_1(5-9) with **(a)** low formation energy E_f, small distortion; **(b)** high E_f, medium distortion; and **(c)** medium E_f, large distortion. Strains are calculated with the reference to the equilibrium bond length (1.42 Å). Positive values denote tensile strains.

Figure S2 illustrates the distributions of bond strains in the presence of different V_1(5-9). Strains around the V_1(5-9) with low E_f and prominent anti-bonding states (**Figure 6(b)**) are slightly larger and more localised. In **Figure S2(b)(c)**, compressive strains

concentrate on the pentagon side of V_1(5-9), while tensile strains dissipate along the direction of the Jahn-Teller reconstructed bond. Comparatively, V_1(5-9) in **Figure S2(a)** does not dramatically change the original strain distribution (**Figure 2(b)**). Defect induced distortions are confined around it. Side views in **Figure S3** also illustrate this point. In **(a)**, the original strain-induced ripples are maintained, while such patterns are largely distorted in **(b)** and **(c)**, which dissipates the local strain and stabilises the V_1(5-9) structure.

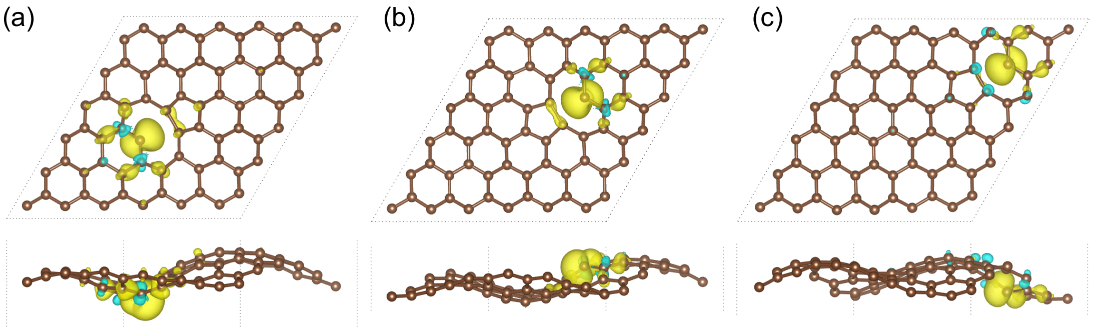

Figure S3 Spin density distributions of distorted V_1(5-9) with **(a)** low E_f, small distortion; **(b)** high E_f, medium distortion; and **(c)** medium E_f, large distortion. Yellow and cyan iso-surfaces correspond to major and minor spins. The value of iso-surfaces is 0.02 $|e|Å^{-3}$.

Figure S3 shows the spin density distributions of different V_1(5-9). Compared with the spin density of V_1(5-9) on freestanding graphene (**Figure 1(c)**), iso-surfaces of 0.02 $|e|Å^{-3}$ are limited around the defect for all the cases, indicating a weakened exchange spin-polarisation effect as V_1(5-9) growing more distorted. From the side views, the electron cloud of the undercoordinated atom displaces vertically, weakening the coupling of its p_z orbital with the π band and increasing sp^3 hybridisation. As the exchange spin-polarisation effect weakens, the unsaturated atom shares the same spin as its nearest neighbours, leading to a different distribution of spin densities in **Figure S3(c)**, corresponding to the curves in **Figure 7**.

Reference

1 M. Hildebrand, F. Abualnaja, Z. Makwana and N. M. Harrison, *J. Phys. Chem. C*, 2019, **123**, 4475–4482.
